# Public knowledge of chronic kidney disease evaluated using a validated questionnaire: a cross-sectional study

**DOI:** 10.1186/s12889-018-5301-4

**Published:** 2018-03-20

**Authors:** Pankti A. Gheewala, Gregory M. Peterson, Syed Tabish R. Zaidi, Matthew D. Jose, Ronald L. Castelino

**Affiliations:** 1 Division of Pharmacy, School of Medicine, Faculty of Health, University of Tasmania, Hobart, TAS Australia; 2 School of Medicine, Faculty of Health, University of Tasmania, Hobart, TAS Australia; 3 Sydney Nursing School, The University of Sydney, Sydney, NSW Australia

**Keywords:** Chronic kidney disease, Cross-sectional study, Public knowledge, Questionnaire, Quiz, Validation

## Abstract

**Background:**

Screening programs may help to address the burden of chronic kidney disease (CKD) in Australia. Public awareness is an important determinant of the uptake of screening programs. However, data on the public knowledge of CKD in Australia is lacking. The aim of this study was to develop a validated questionnaire and assess the Australian public knowledge of CKD.

**Methods:**

A CKD knowledge questionnaire was developed after reviewing the literature and discussions with nephrology experts. Content validity was performed by nephrologists (*n* = 3), renal nurses (*n* = 3) and research personnel (*n* = 4). The questionnaire was piloted in 121 public participants. Next, discriminant validation was performed by recruiting two additional groups of participants: final year undergraduate pharmacy students (*n* = 28) and nephrologists (*n* = 27). Reliability of the questionnaire was assessed by calculating Cronbach’s alpha. Next, a cross-sectional survey of the Australian public (*n* = 943) was conducted by using the validated questionnaire. It was administered using an online Omnibus survey. Quota sampling was used for participant selection and to ensure that the final sample would match the key characteristics of the Australian population. Finally, a standard multiple regression analysis was performed to identify predictors of the public knowledge.

**Results:**

The median CKD knowledge scores of the public, students and nephrologists were 12, 19 and 23 (maximum score of 24), respectively, with statistically significant differences in the scores across the three groups (*p* < 0.001; Kruskal-Wallis test). The Cronbach’s alpha was 0.88 (95% CI: 0.86–0.91), indicating that the questionnaire had good internal consistency. In the cross-sectional survey of the Australian public, the participants’ mean (SD) age was 47.6 (±16.6) years and 51.2% were female. The mean (SD) knowledge score was 10.3 (± 5.0). The multivariate analysis showed that participants with a higher level of education; with a family history of kidney failure; with a personal history of diabetes; and currently or previously living in a relationship had significantly higher knowledge scores.

**Conclusion:**

The Australian public knowledge of CKD was relatively poor. Improving public knowledge may assist in increasing early detection and subsequent management of CKD in Australia.

**Electronic supplementary material:**

The online version of this article (10.1186/s12889-018-5301-4) contains supplementary material, which is available to authorized users.

## Background

Chronic kidney disease (CKD) refers to decreased kidney function, as shown by a glomerular filtration rate (GFR) of less than 60 mL/min per 1·73 m^2^, or markers of kidney damage, or both, of at least 3 months duration [1, 2]. CKD is a major risk factor for end-stage kidney disease, cardiovascular disease and premature death [3]. The global burden of CKD has increased significantly, causing > 500,000 deaths since 1990 [4, 5]. Between 2005 and 2013, the global age-standardised mortality rate for CKD has increased by approximately 37% [4]. Despite this, CKD has received relatively limited global attention and needs effective public health interventions for prevention and management [4].

Early detection and treatment of CKD in its initial stages may help in the prevention or delaying of disease progression [6]. Many clinical practice guidelines for CKD, such as Kidney Health Australia (KHA) - Caring for Australasians with Renal Impairment (CARI), recommend screening of people with risk factors for CKD [6–8] and several screening programs have been conducted worldwide to identify people with early stages of CKD within the community [9]. A screening program conducted within the Australian community setting concluded that implementation of targeted ‘opportunistic’ screening for CKD within primary care might be a sustainable approach [10], while public screening for CKD is not routinely practiced at present in Australia [11].

More generally, health promotion and early detection are important strategies adapted by the Australian government within the national health policy to address increasing rates of chronic diseases [12]. However, there appears to be a lack of understanding amongst the Australian community about the preventability of major health conditions [13, 14]. An Australian survey, which included items to determine the public’s ability to adopt disease preventive measures and engage in early detection by understanding health alerts in the media and public displays for inoculations and screening, found less than adequate levels of health literacy in approximately 60% of participants [15]. Limited public knowledge of the particular disease itself is another important barrier to the successful implementation of prevention programs [13, 16, 17]. For instance, a cross-sectional survey of Australian adults showed that even amongst subgroups of cohorts with the greatest risk of CKD, the knowledge of CKD risk factors and the recall of kidney function testing were both limited [17].

Public awareness of CKD is an important determinant of the uptake of screening programs [18, 19], which may help to address the CKD burden. Determining the public knowledge of CKD can also provide guidance to medical health professionals, researchers and kidney health organisations when establishing the need for education campaigns. The few studies conducted to assess the public knowledge of CKD, used questionnaires that were not validated [17, 20–22], with no such study performed in the general Australian population. Therefore, the primary aim of this study was to determine knowledge of CKD in the Australian public using a newly developed and validated questionnaire. The secondary aim was to determine potential predictors of CKD knowledge in the Australian public.

## Methods

This study involved two phases: Phase 1) Development and validation (content and discriminant validity) of the CKD knowledge questionnaire, and Phase 2) A cross-sectional survey to evaluate the Australian public knowledge of CKD.

### Phase 1) development and validation

The initial draft of the CKD knowledge questionnaire was generated through literature review of existing public [20–22] and related questionnaires [17, 23–27], following discussions with nephrology and research experts. The questionnaire was divided into 5 sections and included a total of 35 evidence-based questions on the physiology of the kidneys, ‘Kidney Health Check’ [28], risk factors for CKD [29] and signs and symptoms of advanced CKD or kidney failure. Seven control items were added to the questionnaire for methodological validity. The questionnaire was reviewed for content and face validity by nephrologists (*n* = 3), renal nurses (*n* = 3) and research personnel (*n* = 4). For each section, reviewers were asked to evaluate individual items and highlight those that were deemed inappropriate in terms of phrasing and applicability. Consequently, items that would require a clinical level of expertise were deleted, and several items were rephrased so that a layperson could better understand them. The final draft of the questionnaire is provided as an Additional file 1. The questionnaire consisted a total of 24 questions with the multiple-choice options ‘True’, ‘False’ and ‘I don’t know’. Correct responses were given a score of 1 and incorrect responses were given a score of 0. The option ‘I don’t know’ was considered as lack of knowledge and given a score of 0.

Next, the questionnaire was piloted and involved recruitment of eligible people visiting the central shopping district and a suburban shopping centre in Hobart, Tasmania. Eligible people were adults (≥18 years) who were not registered healthcare professionals, such as a doctor, nurse, pharmacist or dietitian, and did not have a personal history of kidney failure. A researcher visually determined the eligibility of a potential participant and approached them for participation. Those willing to participate (after confirmation of eligibility) were required to complete the questionnaire on the spot. The first five participants additionally assessed the questionnaire for clarity, formatting and phrasing. Sample size for this pilot phase was calculated as per the recommendations made by Viechtbaur et al. [30]. To be 99% certain that the pilot study would detect any unforeseen problems e.g. misinterpretations of the questionnaire items with a problem probability of 0.05, at least 90 participants was needed.

To determine the discriminant validity of the questionnaire, two additional groups of participants were recruited: final (fourth) year undergraduate pharmacy students and nephrologists. Pharmacy students from the University of Tasmania were invited to answer the self-administered questionnaire during their regular university tutorial sessions. Nephrologists completed an online questionnaire following recruitment through an advertisement in the Australian and New Zealand Society of Nephrology’s weekly newsletter. No previous data (from a pilot study) was available to perform a statistical power analysis for sample size estimation. Therefore, choosing a large effect size of 0.40, with an alpha = 0.05 and power = 0.80, the projected sample size needed was approximately *N* = 66 (22 per group) for between-group comparisons [31]. It was hypothesised that the average knowledge score would be highest for nephrologists, followed by students and lastly the public.

Reliability of the questionnaire was measured by calculating the Cronbach’s alpha. Next, normality of distributions of the continuous variable was determined using the Shapiro-Wilk test. The Kruskal-Wallis test was used to determine if there were any statistically significant differences between the knowledge scores of the three groups (i.e. public, students and nephrologists). Post-hoc tests were performed using Mann-Whitney U tests to determine which groups were significantly different (*p* < 0.005) from one another.

### Phase 2) cross-sectional survey

The above-validated questionnaire was used to evaluate the public knowledge of CKD. Based on the Phase 1 data, it was estimated that at least 50% of the sample would have a total score of at least 50% of the maximum achievable score on the questionnaire. Using a 5% precision and 99% confidence level, to be 99% sure that the true percentage of the public that would achieve at least 50% score on the questionnaire was between 45% and 55%, 665 eligible participants were needed. The questionnaire was administered using I-view’s (an Australian market and social research data collection agency; http://www.iview.com.au/) online Omnibus service [32]. The Omnibus is conducted over a period of 1 week once fortnightly, and provides a national sample of 1000 adults (≥18 years). I-view’s online panel ‘MyView’ was used as the sampling frame; it consists of approximately 130,000 Australian adults (≥18 years) and overseas visitors staying or intending to stay in Australia for 12 months or more. Quotas were set according to age, gender and geographical locations (divided according to state, and by metropolitan and rural areas) to ensure that the final sample would match the characteristics of the Australian population, as per the Australian Bureau of Statistics (ABS) 2011-census data.

The online Omnibus was conducted between 2nd and 6th November 2016. Respondents who completed the survey in under half of the median survey length were identified as skimmers and excluded. Post-stratification was used to make adjustment to the weights so that the resultant weighted estimates from the sample conform to the Australian population values for age, gender and location. The sample (cross-sectional survey data) joint distribution and population (ABS census 2011 data) joint distribution of all three variables (age range, gender and location) was determined. Post-stratification was performed using the rim weighting method. Rim weighting allows benchmarking sample distributions to that of the population distributions. It is an iterative proportional fitting procedure, where all three variables were simultaneously weighted until a convergence was reached.

After the weighted data was obtained from I-view, participants were excluded if they identified themselves as a healthcare professional (such as a doctor, nurse, pharmacist or dietitian) or had a personal history of kidney disease. Given the large sample size, the central limit theorem holds true, and it was reasonably assumed that the distribution of the total score would be approximately normally distributed [33]. Next, bivariate analyses was performed using one-way ANOVA and independent t-tests, as appropriate, to compare the effect of participants’ sociodemographic characteristics on the CKD knowledge score. A multivariable linear regression model was then constructed to predict the knowledge score based on potential predictor variables (*p* < 0.10, using bivariate analysis), and a standard multiple regression analysis was performed. We confirmed the assumptions of normality, linearity and multicollinearity.

The Statistical Package for the Social Sciences (IBM SPSS Statistics for Windows, Version 23.0 Armonk, NY: IBM Corp. SPSS) and G*Power version 3.1 were used to perform all the statistical analyses.

## Results

### Phase 1) development and validation

Complete responses were received from 27 students, 28 nephrologists and 121 participants from the public, and these were included in the final analysis. These corresponded to over 85% of the students and members of the public approached, while the nephrologists voluntarily accessed the online questionnaire. An additional file shows the percentage of correct responses to individual items on the questionnaire by all three groups [see Additional file 2]. The Cronbach’s alpha was 0.88 (95% CI: 0.86–0.91), indicating that the questionnaire had good internal consistency.

The *p*-value for the Shapiro-Wilk test was < 0.05 for each group, indicating that the data was not normally distributed. Therefore, non-parametric statistical tests were used to perform subsequent analyses. The Kruskal-Wallis test revealed a statistically significant difference in the total score of participants across the three groups (χ^2^ (2, *N* = 176) = 109.7, *p* < 0.001). The median total scores of the nephrologists, students and public were 23, 19 and 12, respectively. Post-hoc comparisons performed between pairs of groups found statistically significant differences between all three groups (*p* < 0.001).

### Phase 2) cross-sectional survey

A total of *N* = 24,662 people were invited to participate in the online Omnibus survey. The survey was closed after 1 week, during which time 2173 people accessed the survey and 1034 provided complete responses (response rate of 4.2%). A total of 73 participants were identified as a registered healthcare professional or had a personal history of kidney disease, and 18 had unclear sociodemographic characteristics; these were excluded. Thus, a sample of 943 participants was included in the final analysis, and Table 1 shows participant characteristics and their comparison with the Australian public. Since this sample was matched to only Australian public values for age, gender and location, there are differences in the proportions under the categories education and country of birth. More specifically, this sample had a higher proportion of Australian-born participants and individuals who were higher degree/postgraduate diploma/bachelor degree holders and diploma/vocational holders.

**Table 1:** Participant characteristics and their comparison with the Australian public

Characteristics	Participants		Australian public	
	N	%	N	%
Total	943	100	16,517,613	100
Age (Mean ± S.D., range)	47.6 ± 16.6, 18–84			
Age range (years)^a^				
18–29	181	19.2	3,533,626	21.4
30–49	347	36.8	6,020,939	36.4
50 +	415	44.0	6,963,048	42.2
Gender^a^				
Female	483	51.2	8,446,803	51.1
Male	460	48.8	8,070,810	48.8
Country of birth				
Australia	727	77.1	10,674,865	64.6
Not Australia	216	22.9	5,842,748	35.4
Education`				
Higher degree or postgraduate diploma/Bachelor degree	299	31.7	3,268,574	19.8
Diploma/Vocational	345	36.6	1,392,191	8.4
Completed highest level of school	178	18.9	2,639,997	16.0
Did not complete highest level of school	121	12.8	472,469	2.9
Occupation				
Professional/Managerial	189	20.0		
Sales/Clerical	147	15.6		
Technical/Skilled	81	8.6		
Unskilled/Labourer	53	5.6		
Other occupations	53	5.6		
Do not work	420	44.5		
Work outside home				
Yes, full-time	331	35.1		
Yes, part-time	192	20.4		
No (Not employed, student, work at home, homemaker, retired, etc.)	420	44.5		
Gross annual income				
Under $50,000	348	36.9		
$50,000 to just under $100,000	281	29.8		
$100,000 and over	208	22.1		
Refused	103	11.2		
Marital status				
Married/Common law, De-facto or Living with a partner	568	60.2		
Single/Never married	227	24.1		
Divorced/Separated/Widowed	148	15.7		
Number of people in the household				
One	186	19.7		
Two	349	37.0		
Three	163	17.3		
Four	172	18.2		
Five or more	73	7.7		
Children under 18 years of age				
Yes	285	30.2		
No	658	69.8		
Area description				
Within a capital city	535	56.7		
Within a major regional city	234	24.8		
Within a rural town or its surrounds	144	15.3		
More than 5 km from the nearest town	30	3.2		
State^a^				
New South Wales	296	31.4	5,316,815	32.2
Victoria	257	27.3	4,149,390	25.1
Queensland	181	19.2	3,278,855	19.8
Western Australia	85	9.0	1,709,692	10.4
South Australia	84	8.9	1,247,852	7.6
Tasmania	24	2.5	381,299	2.3
Australian Capital Territory	13	1.4	277,559	1.7
Northern Territory	3	0.3	153,716	0.9
Other territories	0	0	2433	0.0
Aboriginal or Torres Strait Islander descent				
Yes	15	1.6	10,329	0.1
No	928	98.4	16,507,284	99.9
Family member working as a registered healthcare professional e.g. doctor, nurse, dietician or pharmacist				
Yes	55	5.8		
No	888	94.2		
Family history of kidney failure				
Yes	47	5.0		
No	896	95.0		
Medical condition(s)/illness(es) that require regular medications				
High blood pressure known as hypertension				
Yes	220	23.3		
No	704	74.7		
I don’t know	19	2.0		
Raised blood sugar known as diabetes				
Yes	81	8.6		
No	840	89.1		
I don’t know	22	2.3		
Heart problems such as heart failure or heart attack				
Yes	42	4.5		
No	877	93.0		
I don’t know	24	2.5		
Personal history of stroke				
Yes	18	1.9		
No	900	95.4		
I don’t know	25	2.7		

Blanks indicate that the data for the Australian public was not available

^a^Sample was post-weighted to match only three Australian public characteristics (age, gender and state)

`Variables do not add up to total due to education information inadequately described or not stated by the Australian public

**Fig. 1:**
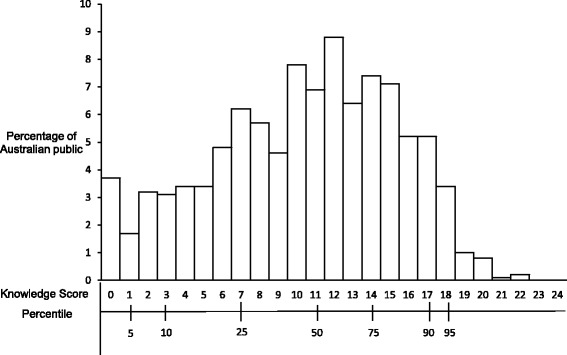
Distribution of the chronic kidney disease knowledge scores of the Australian public

The mean (SD) knowledge score of the Australian public was 10.34 (± 5.0), with values ranging from 0 to 22. As shown in Fig. 1 Distribution of the chronic kidney disease knowledge scores of the Australian public, 50% of the participants had knowledge scores less than 11. Table 2 shows the percentage of participants with correct responses to individual items on the questionnaire. Most participants knew that kidneys make urine (62.1%) and clean blood (69.8%); however, few identified that kidneys help to maintain blood pressure (BP) (26.4%) and keep the bones healthy (14.3%). Many participants identified diabetes (60.6%) as a risk factor, but hypertension (38.3%) was less frequently recognised. Most participants knew that urine (76.2%) and blood (68.2%) tests help to determine the kidney health; however, only 20.3% people knew that BP monitoring also helps in evaluating kidney health. Only 23.4% knew that herbal supplements are not effective in treating CKD and just over 50% knew that medication could help in delaying the progression of CKD.

**Table 2:** Percentage of correct response to individual items on the questionnaire by the Australian general population

Item No	Question	Correct response (%) *N* = 943
1^*^	A person can lead a normal life with one healthy kidney.	85.6
2	Herbal supplements can be effective in treating chronic kidney disease.	23.4
3^*^	Certain medications can help to slow-down the worsening of chronic kidney disease.	51.2
What functions do the kidneys perform in the body?		
4^*^	The kidneys make urine.	62.1
5^*^	The kidneys clean blood.	69.8
6	The kidneys help to keep blood sugar level normal.	22.6
7^*^	The kidneys help to maintain blood pressure.	26.4
8	The kidneys help to breakdown protein in the body.	14.3
9^*^	The kidneys help to keep the bones healthy.	14.3
Which of the following are commonly used to determine health of the kidneys?		
10^*^	A blood test.	68.2
11^*^	A urine test.	76.2
12	A faecal test.	45.9
13^*^	Blood pressure monitoring.	20.3
What are the risk factors for chronic kidney disease?		
14^*^	Diabetes.	60.6
15	Being female.	42.4
16^*^	High blood pressure.	38.3
17^*^	Heart problems such as heart failure or heart attack.	26.3
18	Excess stress.	16.4
19^*^	Obesity.	58.6
What are the signs and symptoms that a person might have if they have advanced chronic kidney disease or kidney failure?		
20^*^	Water retention. (excess water in the body)	61.1
21	Fever.	15.2
22^*^	Nausea/vomiting.	37.6
23^*^	Loss of appetite.	38.4
24^*^	Increased fatigue (tiredness).	58.7

^**a**^ True items

Results of the bivariate analysis performed using one-way ANOVA tests between individual participant characteristics and total score is shown in an Additional file 3. The analysis of variance showed significant associations between the CKD knowledge score and sociodemographic variables, such as age, education, occupation, annual income and marital status, and a personal history of hypertension, diabetes, heart disease and stroke (*p* < 0.01). An additional file shows the results of the bivariate analysis performed using independent t-tests between individual participant characteristic and total score [see Additional file 4]. There was a significant difference (*p* < 0.01) in the knowledge scores of participants with and without a family history of kidney failure.

A multiple linear regression was performed to predict the CKD knowledge score based on age, education, occupation, marital status, family history of kidney failure, and a personal history of hypertension, diabetes, heart disease and stroke. The bivariate analysis showed that participants who refused to reveal their annual income had statistically significantly lower knowledge scores than the other category participants. This participant characteristic was excluded because practically it would not have made a unique contribution to a model that could be used to predict knowledge scores. Table 3 shows the results of the standard multiple regression analysis between CKD knowledge score and participant characteristics. A significant regression equation was found (F (21,921) = 4.58, *p* < 0.001), with an R^2^ of 0.095. The multivariate analysis found higher knowledge scores associated with a higher level of education, such as possessing a postgraduate diploma or bachelor degree and diploma/vocational certificate. A family history of kidney failure was also independently associated with higher knowledge scores, as was a personal history of diabetes. Finally, participants currently or previously within a relationship (married, de-facto, living with a partner or divorced/separated/widowed) had significantly higher knowledge scores than those who were single/never married.

**Table 3:** Standard multiple regression analysis between CKD knowledge score and participant characteristics

Characteristics	β coefficient (95% CI)	*p* value
Age range (years)		
18 – 29^a^		
30–49	−0.06 (−1.56 to 0.25)	0.16
50 +	0.04 (−0.57 to 1.43)	0.40
Education		
Did not complete highest level of school^a^		
Completed highest level of school	0.01 (−0.99 to 1.28)	0.80
Diploma/Vocational	0.11 (0.10 to 2.14)	**0.03**
Higher degree or post graduate diploma/Bachelor degree	0.16 (0.58 to 2.79)	**0.003**
Occupation		
Do not work^a^		
Unskilled/Labourer	−0.04 (−2.35 to 0.43)	0.18
Technical/Skilled	−0.01 (−1.27 to 1.10)	0.89
Sales/Clerical	−0.02 (− 1.23 to 0.67)	0.57
Professional/Managerial	0.06 (−0.32 to 1.64)	0.11
Other occupations	0.05 (−0.39 to 2.43)	0.16
Marital status		
Single/Never married^a^		
Married/Common law, De-facto or Living with a partner	0.10 (0.21 to 1.80)	**0.01**
Divorced/Separated/Widowed	0.10 (0.28 to 2.48)	**0.01**
Family history of kidney failure		
Yes vs No	0.08 (0.39 to 3.24)	**0.01**
Medical condition(s)/illness(es) that require regular medications		
High blood pressure known as hypertension		
Yes^a^		
No	−0.02 (−1.09 to 0.55)	0.52
I don’t know	−0.09 (−6.84 to 0.29)	0.07
Raised blood sugar known as diabetes		
Yes^a^		
No	−0.09 (−2.54 to − 0.22)	**0.02**
I don’t know	−0.07 (−5.37 to 0.79)	0.15
Heart problems such as heart failure or heart attack		
Yes^a^		
No	0.01 (−1.29 to 1.86)	0.73
I don’t know	0.03 (−2.31 to 4.22)	0.57
Personal history of stroke		
Yes^a^		
No	−0.09 (−4.32 to 0.27)	0.08
I don’t know	−0.16 (−8.43 to −1.15)	**0.01**

^a^Reference; R^2^ for the model = 0.095

Bold numbers indicate *p*-values which were found to be statistically significant in the multivaraiate analysis

## Discussion

Overall, the results of this study show poor understanding of CKD amongst the Australian public. Participants in this study had limited knowledge of the physiological role of the kidneys, especially relating to the regulation of BP, and bone development and metabolism. Participant knowledge about CKD risk factors was also limited. Less than half of the participants correctly identified hypertension as a risk factor. This percentage was higher, however, than the 2.8% reported in a study of 852 Australians by White et al. [17] In a public survey of 748 participants conducted in Iran [21], only 14.4% selected ‘unmanaged hypertension’ as ‘very likely to result in CKD’; whereas, a study of 516 community-dwelling Hong Kong adults reported that 43.8% participants knew that hypertension can cause kidney disease [20]. Additionally, a cross-sectional study of 454 participants conducted in South-West Nigeria found that 54.7% believed that hypertension was a CKD risk factor [22]. Conversely, the percentage of participants (60.6%) who correctly identified diabetes as a risk factor in this study was high as compared to the 8.6%, 12.7%, 44.0% and 49.0% reported by White et al. [17] Roomizadeh et al. [21], Chow et al. [20] and Oluyombo et al. [22], respectively.

Only half of the participants knew that medications can help to slow the worsening of CKD. This suggests that the Australian public understanding of the treatment of kidney failure is relatively poor. In addition, only 23.4% of participants knew that herbal supplements are ineffective in treating CKD. Some herbal supplements have been associated with the development of CKD [34] and related to acute kidney injury [35]. Also, concomitant use of herbs and conventional drugs can cause drug toxicity and therapeutic failure via alteration in renal function [36]. With the increasing availability and use of herbal medicines in high-income countries [37], efforts should be made to educate people on their potentially harmful repercussions, and their use should be strongly discouraged in people with kidney disease.

KHA recommends that people with diabetes or hypertension should undergo a ‘Kidney Health Check’ every year [28]. A ‘Kidney Health Check’ includes three assessments: a blood test to determine the estimated GFR (indicates level of kidney function); a urine test to check for albuminuria (marker of kidney damage); and an assessment of BP because kidney disease can be an outcome of high BP or cause renal hypertension. More than 65% of participants in this study knew that blood and urine tests can be used to determine kidney health; however, few correctly identified a BP assessment. Both hypertension and CKD are silent diseases, which warrant regular monitoring for prevention and management. There is a need to create improved awareness about BP, its regular monitoring and its association with CKD amongst the Australian public.

The multivariate analysis showed that CKD knowledge score increased with a higher level of education; this is consistent with the findings of other studies [20, 22]. Additionally, our regression model showed that participants who were single or never married had lower CKD knowledge scores. This may be because people who have lived or are living with others are more actively involved in acquiring health-related information and implementing healthy lifestyles [38–41]. Another important predictor variable was a family history of kidney failure. This was an expected outcome because knowing a person with kidney failure would be anticipated to indirectly raise awareness on the same.

White et al. [17] and Chow et al. [20] found, similar to this study, that participants with a personal history of diabetes had better knowledge. Although this predictor variable reached statistical significance in the final model, the mean total score of patients with diabetes was low (11.8 out of a possible score of 24). This suggests that even though patients with diabetes had better knowledge when compared with the public, their overall CKD knowledge was still poor. Similarly, even though participants with other existing co-morbidities had comparatively higher mean scores, the values were still around half of the maximum achievable on the questionnaire. This demonstrates that even amongst the cohorts at highest risk of developing CKD, awareness is relatively low. The KHA-CARI guidelines recommend that physicians should provide early CKD education to patients with CKD risk factors as this may prevent CKD development and progression [6]. A recent Australian study conducted to determine the kidney disease health-literacy among new patients referred to specialist nephrology care reported that 35.8% patients had received no education and 46.2% had little, but inadequate, information on their kidney problem [25]. When asked what causes CKD, almost 40% patients answered ‘unsure’ and approximately 30% answered ‘alcohol’.

Some inconsistencies were found when comparing the questionnaire results of this study with those of others. These, in part, may be because of the exploratory nature of the questions and non-validated questionnaires used in other studies. Creating a questionnaire that can produce valid and reliable data is a complex process, and guidelines are available for developing and validating questionnaires before their use in cross-sectional studies [42–44]. Despite this, studies have often used non-validated questionnaires [42]. Prior to the future use of this questionnaire, several improvements that could be made include: 1) Rephrasing section 2 as “What major functions do the kidney perform in our body?” (noting that there is some involvement in controlling blood glucose levels) and 2) Addition of an item under Section 2 “Kidneys help in the production of red blood cells” (True Item).

It is acknowledged that the sample may not have been truly representative of the general public. It was weighted to match the Australian population for only age, gender and location, and had a relatively high proportion of participants who were higher degree/postgraduate diploma/bachelor and diploma/vocational degree holders. Also, more than 20% of the participants had a gross annual income of $100,000 and over. Despite this, the mean total score of participants was less than 50% of the maximum score achievable on the questionnaire. This suggests that everyone should be targeted for CKD education, irrespective of their sociodemographic backgrounds. While the regression model was statistically significant, the R square value was low. Hence, future studies should explore additional predictors, which can further assist in understanding the low CKD knowledge of the public.

## Conclusions

A valid and reliable questionnaire to measure the CKD knowledge of the general population was developed and tested in this study. Australian public knowledge of the physiological role of the kidneys, and CKD and its risk factors was poor, irrespective of sociodemographic and clinical characteristics. Healthcare professionals within primary care settings should evaluate the CKD knowledge of patients with CKD risk factors and, if warranted, provide tailored education. As for the public, there is a need to increase their understanding of kidneys and knowledge of CKD through nationwide awareness programs. These efforts may improve the early detection and management of CKD.

## Additional files

Additional file 1: Chronic Kidney Disease Knowledge Questionnaire, This file includes the original questionnaire used to evaluate the Australian public knowledge about chronic kidney disease. (DOCX 17 kb)

Additional file 2: Percentage of correct response to individual items on the questionnaire. This files includes Phase 1 data on the percentage of correct response to individual items on the questionnaire provided by the nephrologists, students and public. (DOCX 15 kb)

Additional file 3: Results of the bivariate analysis performed using one-way ANOVA test between individual participant characteristics and total score. This files includes Phase 2 data on the bivariate analysis that was performed using one-way ANOVA test. This data shows which sociodemographic characteristics of the Australian public were significantly statistically associated with the total knowledge score. (DOCX 18 kb)

Additional file 4: Results of the bivariate analysis performed using Independent t-test between individual participant characteristic and total score. This files includes Phase 2 data on the bivariate analysis that was performed using Independent t-test and details the statistically significant associations between various sociodemographic characteristics of the Australian public and the total CKD knowledge score. (DOCX 14 kb)

## Abbreviations

ABS: Australian Bureau of Statistics
CKD: Chronic kidney disease
GFR: Glomerular filtration rate
KHA-CARI: Kidney Health Australia - Caring for Australasians with Renal Impairment
