# Supplementary material for: Public knowledge of chronic kidney disease evaluated using a validated questionnaire: a cross-sectional study
Source: BMC Public Health. 2018 Mar 20;18:371. doi: 10.1186/s12889-018-5301-4 (PMC5859642; doi:10.1186/s12889-018-5301-4)
Supplement: Supplementary file 1 — Chronic Kidney Disease Knowledge Questionnaire, This file includes the original questionnaire used to evaluate the Australian public knowledge about chronic kidney disease. (DOCX 17 kb) [file 12889_2018_5301_MOESM1_ESM.docx]

**Chronic Kidney Disease Knowledge Questionnaire**

**For Sections, 1-5, please answer ‘True’, ‘False’ or ‘I don’t know’ to the following questions:**

**Section 1**

| **No** | **Question** | **True** | **False** | **I don’t know** |
| --- | --- | --- | --- | --- |
| 1 | A person can lead a normal life with one healthy kidney. | □ | □ | □ |
| 2 | Herbal supplements can be effective in treating chronic kidney disease. | □ | □ | □ |
| 3 | Certain medications can help to slow-down the worsening of chronic kidney disease. | □ | □ | □ |

**Section 2** What functions do the kidney perform in our body?

| **No** | **Question** | **True** | **False** | **I don’t know** |
| --- | --- | --- | --- | --- |
| 4 | The kidneys make urine. | □ | □ | □ |
| 5 | The kidneys clean blood. | □ | □ | □ |
| 6 | The kidneys help to keep blood sugar level normal. | □ | □ | □ |
| 7 | The kidneys help to maintain blood pressure. | □ | □ | □ |
| 8 | The kidneys help to breakdown protein in the body. | □ | □ | □ |
| 9 | The kidneys help to keep the bones healthy. | □ | □ | □ |

**Section 3** Which of the following are commonly used to determine the health of your kidneys?

| **No** | **Question** | **True** | **False** | **I don’t know** |
| --- | --- | --- | --- | --- |
| 10 | A blood test. | □ | □ | □ |
| 11 | A urine test. | □ | □ | □ |
| 12 | A faecal (poo) test. | □ | □ | □ |
| 13 | Blood pressure monitoring. | □ | □ | □ |

**Section 4** What are the risk factors for chronic kidney disease?

| **No** | **Question** | **True** | **False** | **I don’t know** |
| --- | --- | --- | --- | --- |
| 14 | Diabetes. | □ | □ | □ |
| 15 | Being female. | □ | □ | □ |
| 16 | High blood pressure. | □ | □ | □ |
| 17 | Heart problems such as heart failure or heart attack. | □ | □ | □ |
| 18 | Excess stress. | □ | □ | □ |
| 19 | Obesity. | □ | □ | □ |

**Section 5** What are the signs and symptoms that a person might have if they have advanced chronic kidney disease or kidney failure?

| **No** | **Question** | **True** | **False** | **I don’t know** |
| --- | --- | --- | --- | --- |
| 20 | Water retention (excess water in the body). | □ | □ | □ |
| 21 | Fever. | □ | □ | □ |
| 22 | Nausea/vomiting. | □ | □ | □ |
| 23 | Loss of appetite. | □ | □ | □ |
| 24 | Increased fatigue (tiredness). | □ | □ | □ |

**Thank you very much for your time and participation in this questionnaire**
