# Supplementary material for: Public knowledge of chronic kidney disease evaluated using a validated questionnaire: a cross-sectional study
Source: BMC Public Health. 2018 Mar 20;18:371. doi: 10.1186/s12889-018-5301-4 (PMC5859642; doi:10.1186/s12889-018-5301-4)
Supplement: Supplementary file 2 — Percentage of correct response to individual items on the questionnaire. This files includes Phase 1 data on the percentage of correct response to individual items on the questionnaire provided by the nephrologists, students and public. (DOCX 15 kb) [file 12889_2018_5301_MOESM2_ESM.docx]

**Additional file 2** Percentage of correct response to individual items on the questionnaire

| Item No | Question | Correct response (%) | | |
| --- | --- | --- | --- | --- |
|  |  | General public (n=121) | Student  (n=28) | Nephrologist (n=27) |
| 1* | A person can lead a normal life with one healthy kidney. | 94.2 | 96.4 | 100.0 |
| 2 | Herbal supplements can be effective in treating chronic kidney disease. | 27.3 | 82.1 | 77.8 |
| 3* | Certain medications can help to slow-down the worsening of chronic kidney disease. | 71.9 | 96.4 | 100.0 |
| What functions do the kidneys perform in the body? | | | | |
| 4* | The kidneys make urine. | 57.0 | 96.4 | 100.0 |
| 5* | The kidneys clean blood. | 68.6 | 82.1 | 100.0 |
| 6 | The kidneys help to keep blood sugar level normal. | 18.2 | 50.0 | 85.2 |
| 7* | The kidneys help to maintain blood pressure. | 24.8 | 100.0 | 100.0 |
| 8 | The kidneys help to breakdown protein in the body. | 17.4 | 57.1 | 81.5 |
| 9* | The kidneys help to keep the bones healthy. | 15.7 | 64.3 | 100.0 |
| Which of the following are commonly used to determine health of the kidneys? | | | | |
| 10* | A blood test. | 77.7 | 96.4 | 100.0 |
| 11* | A urine test. | 82.6 | 96.4 | 100.0 |
| 12 | A faecal (poo) test. | 49.6 | 89.3 | 96.3 |
| 13* | Blood pressure monitoring. | 26.4 | 57.1 | 92.6 |
| What are the risk factors for chronic kidney disease? | | | | |
| 14* | Diabetes. | 71.9 | 100.0 | 100.0 |
| 15 | Being female. | 39.7 | 57.1 | 88.9 |
| 16* | High blood pressure. | 52.9 | 100.0 | 100.0 |
| 17* | Heart problems such as heart failure or heart attack. | 28.9 | 89.3 | 81.5 |
| 18 | Excess stress. | 13.2 | 35.7 | 81.5 |
| 19* | Obesity. | 74.4 | 89.3 | 96.3 |
| What are the signs and symptoms that a person might have if they have advanced chronic kidney disease or kidney failure? | | | | |
| 20* | Water retention. (excess water in the body) | 72.7 | 82.1 | 96.3 |
| 21 | Fever. | 16.5 | 53.6 | 100.0 |
| 22* | Nausea/vomiting. | 49.6 | 64.3 | 96.3 |
| 23* | Loss of appetite. | 43.0 | 89.3 | 96.3 |
| 24* | Increased fatigue (tiredness). | 76.0 | 100.0 | 100.0 |

********True items.*
