# Supplementary material for: Public knowledge of chronic kidney disease evaluated using a validated questionnaire: a cross-sectional study
Source: BMC Public Health. 2018 Mar 20;18:371. doi: 10.1186/s12889-018-5301-4 (PMC5859642; doi:10.1186/s12889-018-5301-4)
Supplement: Supplementary file 3 — Results of the bivariate analysis performed using one-way ANOVA test between individual participant characteristics and total score. This files includes Phase 2 data on the bivariate analysis that was performed using one-way ANOVA test. This data shows which sociodemographic characteristics of the Australian public were significantly statistically associated with the total knowledge score. (DOCX 18 kb) [file 12889_2018_5301_MOESM3_ESM.docx]

**Additional file 3** Results of the bivariate analysis performed using one-way ANOVA test between individual participant characteristics and total score

|  | | Total score mean (SD) | | Df^a^ | | F | | p-value | | Eta-squared^c^ | Post-hoc comparison ^b^ | |
| --- | --- | --- | --- | --- | --- | --- | --- | --- | --- | --- | --- | --- |
| *Total* | | *10.34 (5.0)* | |  | |  | |  | |  |  | |
| *Age range (years)* | |  | | 2, 940 | | 6.7 | | **<0.005** | | 0.01 | 50 + > 18 – 29, 30 – 49 | |
| 18 – 29 | | 9.9 (5.5)* | |  | |  | |  | |  |  |  |
| 30 – 49 | | 9.8 (5.1)* | |  | |  | |  | |  |  |  |
| 50 + | | 11.0 (4.5)* | |  | |  | |  | |  |  |  |
| *Education* | |  | | 3, 939 | | 4.7 | | **<0.005** | | 0.01 | Higher degree or post diploma/Bachelor degree > Completed highest level of school | |
| Higher degree or post graduate diploma/Bachelor degree | | 11.0 (5.0)* | |  | |  | |  | |  |  |  |
| Diploma/Vocational | | 10.5 (4.8) | |  | |  | |  | |  |  |  |
| Completed highest level of school | | 9.4 (5.3)* | |  | |  | |  | |  |  |  |
| Did not complete highest level of school | | 9.7 (4.7) | |  | |  | |  | |  |  |  |
| *Occupation* | |  | | 5, 937 | | 3.3 | | **<0.01** | | 0.02 | Professional/ Managerial > Sales/Clerical, Unskilled/Labourer | |
| Professional/Managerial | | 11.2 (4.7)* | |  | |  | |  | |  |  |  |
| Sales/Clerical | | 9.4 (5.4)* | |  | |  | |  | |  |  |  |
| Technical/Skilled | | 10.0 (5.2) | |  | |  | |  | |  |  |  |
| Unskilled/Labourer | | 8.9 (5.3)* | |  | |  | |  | |  |  |  |
| Other occupations | | 10.8 (5.0) | |  | |  | |  | |  |  |  |
| Do not work | | 10.4 (4.8) | |  | |  | |  | |  |  |  |
| *Work outside home* | |  | | 2, 940 | | 0.2 | | 0.815 | | NA | NA | |
| Yes, full-time | | 10.2 (5.2) | |  | |  | |  | |  |  |  |
| Yes, part-time | | 10.3 (5.1) | |  | |  | |  | |  |  |  |
| No (Not employed, student, work at home, homemaker, retired, etc.) | | 10.4 (4.8) | |  | |  | |  | |  |  |  |
| *Gross annual income* | |  | | 3, 939 | | 5.3 | | <0.005 | | 0.02 | Refused < Under $50,000, $50,000 to just under $100,000, $100,000 and over | |
| Under $50,000 | | 10.3 (4.9)* | |  | |  | |  | |  |  |  |
| $50,000 to just under $100,000 | | 10.6 (4.9)* | |  | |  | |  | |  |  |  |
| $100,000 and over | | 10.9 (5.1)* | |  | |  | |  | |  |  |  |
| Refused | | 8.6 (5.1)* | |  | |  | |  | |  |  |  |
| *Marital status* | |  | | 2, 940 | | 7.0 | | **<0.005** | | 0.01 | Single/Never married < Married/Common law, De-facto or Living with a partner, Divorced/Separated/Widowed | |
| Married/Common law, De-facto or Living with a partner | | 10.5 (4.7)* | |  | |  | |  | |  |  |  |
| Single/Never married | | 9.3 (5.6)* | |  | |  | |  | |  |  |  |
| Divorced/Separated/Widowed | | 11.1 (4.7)* | |  | |  | |  | |  |  |  |
| *Number of people in the household* | |  | | 4, 938 | | 1.3 | | 0.265 | | NA | NA | |
| One | | 10.2 (5.2) | |  | |  | |  | |  |  |  |
| Two | | 10.6 (4.9) | |  | |  | |  | |  |  |  |
| Three | | 10.8 (4.8) | |  | |  | |  | |  |  |  |
| Four | | 10.0 (5.0) | |  | |  | |  | |  |  |  |
| Five or more | | 9.4 (4.9) | |  | |  | |  | |  |  |  |
| *Area description* |  | | 3, 939 | | 1.2 | | 0.306 | | NA | | NA |  |
| Within a capital city | 10.4 (5.0) | |  | |  | |  | |  | |  |  |
| Within a major regional city | 10.2 (4.9) | |  | |  | |  | |  | |  |  |
| Within a rural town or its surrounds | 10.7 (4.8) | |  | |  | |  | |  | |  |  |
| More than 5km from the nearest town | 8.8 (5.4) | |  | |  | |  | |  | |  |  |
| *Location* |  | | 7,935 | | 0.6 | | 0.789 | | NA | | NA |  |
| Victoria | 10.3 (4.9) | |  | |  | |  | |  | |  |  |
| Western Australia | 10.4 (5.2) | |  | |  | |  | |  | |  |  |
| Tasmania | 10.2 (4.3) | |  | |  | |  | |  | |  |  |
| ACT | 11.8 (4.1) | |  | |  | |  | |  | |  |  |
| Northern Territory | 9.7 (1.5) | |  | |  | |  | |  | |  |  |
| NSW | 10.6 (5.1) | |  | |  | |  | |  | |  |  |
| Queensland | 10.1 (4.9) | |  | |  | |  | |  | |  |  |
| South Australia | 9.8 (4.8) | |  | |  | |  | |  | |  |  |
| Do you have any of the following medical condition(s)/illness (es) that require you to take regular medications? | | | | | | | | | | | |  |
| *High blood pressure known as hypertension* |  | | 2, 940 | | 12.3 | | **<0.001** | | 0.03 | | I don’t know < Yes, No |  |
| Yes | 11.1 (4.4)* | |  | |  | |  | |  | |  |  |
| No | 10.3 (5.1)* | |  | |  | |  | |  | |  |  |
| I don’t know | 5.4 (5.6)* | |  | |  | |  | |  | |  |  |
| *Raised blood sugar known as diabetes* |  | | 2, 940 | | 12.0 | | **<0.001** | | 0.02 | | Yes > I don’t know, No;  No > I don’t know |  |
| Yes | 11.8 (4.0)* | |  | |  | |  | |  | |  |  |
| No | 10.3 (5.0)* | |  | |  | |  | |  | |  |  |
| I don’t know | 6.1 (5.6)* | |  | |  | |  | |  | |  |  |
| *Heart problems such as heart failure or heart attack* |  | | 2, 940 | | 6.0 | | **<0.005** | | 0.01 | | I don’t know < Yes, No |  |
| Yes | 10.9 (4.8)* | |  | |  | |  | |  | |  |  |
| No | 10.4 (4.9)* | |  | |  | |  | |  | |  |  |
| I don’t know | 6.9 (5.9)* | |  | |  | |  | |  | |  |  |
| *Personal history of stroke* |  | | 2, 940 | | 11.6 | | **<0.001** | | 0.02 | | I don’t know < Yes, No |  |
| Yes | 12.8 (4.6)* | |  | |  | |  | |  | |  |  |
| No | 10.4 (4.9)* | |  | |  | |  | |  | |  |  |
| I don’t know | 6.1 (5.6)* | |  | |  | |  | |  | |  |  |

^a^ df values (Between groups, Within groups)

^b^ Tukey HSD post hoc comparison (*p<0.05)*

*, Bold indicates variable with a statistical significance that will be included in the multiple linear regression model

^c^ Cohen classifies Eta-squared value of 0.01 as a small effect, 0.06 as a medium effect and 0.14 as a large effect.
