# Supplementary material for: Public knowledge of chronic kidney disease evaluated using a validated questionnaire: a cross-sectional study
Source: BMC Public Health. 2018 Mar 20;18:371. doi: 10.1186/s12889-018-5301-4 (PMC5859642; doi:10.1186/s12889-018-5301-4)
Supplement: Supplementary file 4 — Results of the bivariate analysis performed using Independent t-test between individual participant characteristic and total score. This files includes Phase 2 data on the bivariate analysis that was performed using Independent t-test and details the statistically significant associations between various sociodemographic characteristics of the Australian public and the total CKD knowledge score. (DOCX 14 kb) [file 12889_2018_5301_MOESM4_ESM.docx]

**Additional file 4** Results of the bivariate analysis performed using Independent t-test between individual participant characteristic and total score.

|  | *Total score Mean (SD)* | *t* | *df* | *p-value* | *Eta-squared^a^* |
| --- | --- | --- | --- | --- | --- |
| *Gender* |  | 1.19 | 941 | 0.24 |  |
| Female | 10.5 (4.9) |  |  |  |  |
| Male | 10.1 (5.0) |  |  |  |  |
| *Country of birth* |  | -1.19 | 941 | 0.23 |  |
| Australia | 10.5 (4.9) |  |  |  |  |
| Not Australia | 10.0 (5.1) |  |  |  |  |
| *Children under 18 years of age* |  | -0.08 | 941 | 0.93 |  |
| Yes | 10.3 (4.9) |  |  |  |  |
| No | 10.4 (5.0) |  |  |  |  |
| Are you of Aboriginal or Torres Strait Islander descent? |  | -0.56 | 941 | 0.56 |  |
| Yes | 9.6 (5.4) |  |  |  |  |
| No | 10.4 (5.0) |  |  |  |  |
| Does anyone in your immediate family work as a registered healthcare professional e.g. doctor, nurse, dietician or pharmacist? |  | 1.32 | 941 | 0.19 |  |
| Yes | 11.2 (4.5) |  |  |  |  |
| No | 10.3 (5.0) |  |  |  |  |
| Do you have a family history of kidney failure? |  | 3.23 | 941 | **<0.01** | 0.01 |
| Yes | 12.2 (4.6) |  |  |  |  |
| No | 10.2 (5.0) |  |  |  |  |

Bold indicates variable with a statistical significance that will be included in the multiple linear regression model

^a^ Cohen classifies Eta-squared value of 0.01 as a small effect, 0.06 as a medium effect and 0.14 as a large effect.
